# High-order random Raman lasing in a PM fiber with ultimate efficiency and narrow bandwidth

**DOI:** 10.1038/srep22625

**Published:** 2016-03-04

**Authors:** Sergey A. Babin, Ekaterina A. Zlobina, Sergey I. Kablukov, Evgeniy V. Podivilov

**Affiliations:** 1 Institute of Automation and Electrometry SB RAS, Novosibirsk, 630090, Russia; 2 Novosibirsk State University, Novosibirsk, 630090, Russia

## Abstract

Random Raman lasers attract now a great deal of attention as they operate in non-active turbid or transparent scattering media. In the last case, single mode fibers with feedback via Rayleigh backscattering generate a high-quality unidirectional laser beam. However, such fiber lasers have rather poor spectral and polarization properties, worsening with increasing power and Stokes order. Here we demonstrate a linearly-polarized cascaded random Raman lasing in a polarization-maintaining fiber. The quantum efficiency of converting the pump (1.05 μm) into the output radiation is almost independent of the Stokes order, amounting to 79%, 83%, and 77% for the 1^st^ (1.11 μm), 2^nd^ (1.17 μm) and 3^rd^ (1.23 μm) order, respectively, at the polarization extinction ratio >22 dB for all orders. The laser bandwidth grows with increasing order, but it is almost independent of power in the 1–10 W range, amounting to ~1, ~2 and ~3 nm for orders 1–3, respectively. So, the random Raman laser exhibits no degradation of output characteristics with increasing Stokes order. A theory adequately describing the unique laser features has been developed. Thus, a full picture of the cascaded random Raman lasing in fibers is shown.

Random lasers represent now a rapidly growing class of light sources, in which a conventional optical cavity is substituted by a multiple-scattering feedback in a disordered gain medium, such as laser-crystal or semiconductor powders, see^1^,^2^ for a review. Recent developments in this field include improvements of the random laser performances, as well as demonstrations of lasing in disordered media of new types. So, low-threshold surface-plasmon-enhanced lasing is demonstrated in a matrix of randomly distributed gold nano-islands coated by a wave-guiding layer of a dye-doped polymer^3^ or in a semiconductor active medium (ZnO nanorods) with graphene oxide nanoflakes^4^. Fluidic paper-based random laser devices are fabricated by conventional soft-lithography techniques on a usual paper^5^. Random lasing may be obtained in such exotic media as cold-vapor atoms^6^, or biological tissues including active dye-infiltrated bones^7^, butterfly wing with semiconductor ZnO nanoparticles^8^, and even a single cell^9^. These results initiate the development of advanced technologies toward the realization of biocompatible and implantable active photonic components^8^,^9^, bio-imaging of a new type including mapping of malignant tumors^10^, diagnostics/dynamics of granular^11^ or turbid^12^ media with a great potential in pharmacology, as well as the development of low-coherence sources suitable for speckle-free full-field microscopy or digital light projector systems^13^.

For the development of new light sources, a competitive device performance becomes quite a challenge. In this sense, fiber-based random lasers^14^ are recognized as light sources superior to random lasers of other types, and in some cases to conventional lasers. The fiber waveguide structure is nearly one-dimensional forming an output beam of high quality (single transverse mode with a Gaussian beam profile) in a desired direction by using fiber flexibility. For random lasing, even conventional telecom fibers are suitable. As the fiber material (silica glass) is highly transparent for radiation, especially in the telecom spectral window around 1.5 μm, the gain and feedback mechanisms here are quite different from those in bulk random lasers. The fiber gain is induced by inelastic stimulated Raman scattering (SRS) of the pump light by vibrating SiO_2_ molecules in a glass lattice, whereas the feedback is provided by elastic Rayleigh scattering of the SRS-induced Stokes wave on sub-micron irregularities of the glass structure, with a small part (~10^−3^) of scattered light coming back into the fiber. Though the feedback is very weak, it is sufficient for lasing in a kilometers-long passive fiber given that the integral Raman gain is proportional to the fiber length and pump power.

As shown recently^12^, a high-power non-resonant pumping enables Raman lasing in non-active bulk random materials (e.g. BaSO_4_^12^) as well, which makes random lasing possible in almost any “white” powder, thus offering a new direction in the development of devices and diagnostic techniques. Nevertheless, fiber-based random Raman lasers demonstrate at the moment the highest efficiency of pump-to-Stokes wave conversion exceeding 70% both for the first^15^,^16^,^17^ and for the second Stokes order^18^, with the output beam power up to 200 W^19^. Such random Raman fiber lasers (RRFLs) generate a quasi-continuous mode-free spectrum with the resulting shape defined by the Schawlow-Townes narrowing near the threshold and nonlinear broadening at high powers^14^,^20^. Fiber-based spectral filters can be relatively simply imbedded into the low-power part of RRFLs providing flat tuning within the entire Raman gain spectral range of >35 nm^21^, as well as power-equalized multi-wavelength generation^22^, and order-of-magnitude spectral width reduction defined by the filter characteristics^23^. RRFLs also grant configurations/regimes similar to those in conventional fiber lasers, such as direct pumping by inexpensive and powerful multimode diodes^24^, internal intensity modulation^25^, pulsed operation via active^26^ or passive^27^ Q-switching, etc.

Here we report on the first demonstration of high-order random Raman lasing in a polarization-maintaining (PM) fiber enabling linearly-polarized output radiation of extreme stability, efficiency and narrow bandwidth at a power level up to ~10 W. As the generation thresholds of the Stokes components are two times lower than those in depolarized RRFLs, we obtained up to 4th order in only 1-km long fiber. The cascaded generation of the RRFL in the all-fiber PM configuration exhibits no degradation of output characteristics with increasing Stokes order. The unique features of the cascaded RRFL are analyzed within the framework of the developed analytical model. In the studied scheme, all waves have the same linear polarization, which simplifies simulation of power conversion and spectral broadening processes. The obtained formulae predict with a high accuracy the output power and bandwidth as a function of the pump power and Stokes order, which is useful both in fundamental research and practical applications of random fiber lasers.

## Results

### Experiment

RRFLs employing conventional single-mode fibers under depolarized pumping generate depolarized (or randomly polarized) light^14^,^15^,^16^,^17^,^18^,^19^,^20^,^21^,^22^,^23^,^24^,^25^,^26^,^27^. The first attempts to govern the polarization state of the RRFL output radiation faced some problems. Implementation of linearly polarized pumping^28^ in the RRFL scheme results in generation of partially polarized output radiation. The laser characteristics (threshold, output power, efficiency, and degree of polarization) appear to be significantly influenced by the polarization state of pump radiation. Furthermore, the results indicate that the efficiency of lasing is reduced considerably compared to that for depolarized pumping. In another experiment with the so called half-open RRFL configuration comprising the Bragg grating reflector at one side of the fiber^29^, implementation of the PM fiber under depolarized pumping results in predominantly linear polarization with a 14-dB polarization extinction ratio (PER) at the Watt level, but it almost fully degrades (PER < 3 dB) as the generated power approaches 9.5 W in spite of applying special measures such as a strong coiling of fiber for selection of one polarization component. At that, the maximum conversion efficiency is also rather low (~40%). Here we propose and investigate a new scheme of a cascaded random laser based on an all-PM all-fiber configuration with linearly polarized pumping^30^ that does not suffer from the discussed drawbacks.

**Figure 1:**
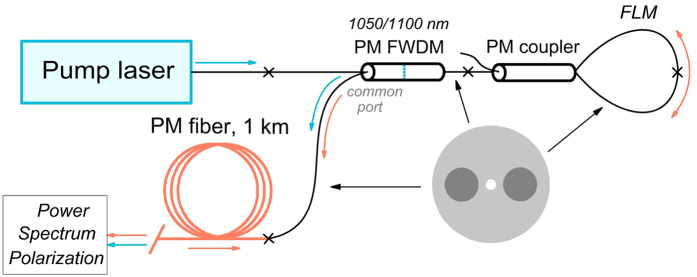
Experimental setup: PM FWDM - polarization maintaining filtered wavelength-division multiplexer with 1050 nm, 1100 nm and common ports; PM coupler – polarization maintaining fused fiber coupler with splitting ratio 50/50 at 1050 nm; FLM - fiber loop mirror.

The experimental setup is schematically shown in Fig. 1. The all-fiber CW pump source is based on a master-oscillator power-amplifier (MOPA) scheme, see Methods section for more details. Pump radiation at 1.05 μm is launched via a 1050-nm port of a high-power 1050/1100 nm PM filtered wavelength division multiplexer (FWDM) having an internal filter reflecting pump radiation into the common port to which a 1 km-long single-mode PM fiber of the Panda type (Fujikura SM98-PS-U25D) is connected. The 1100-nm port of the FWDM is spliced to a PM fiber coupler with coupling ratio of 50/50 at 1.05 μm, which forms a PM fiber loop mirror (FLM) after splicing together its output ports. The FLM reflection coefficient *R* amounts to 91% at 1.11 μm (1^st^ Stokes), 66% at 1.17 μm (2^nd^ Stokes), 36% at 1.23 μm (3^rd^ Stokes), and as low as 12% at 1.3 μm (4^th^ Stokes) in correspondence with coupling ratio of the PM fiber coupler at these wavelengths.

As an output fiber end is cleaved with an angle of >10° to eliminate the Fresnel reflection, the feedback in this scheme is provided by random Rayleigh backscattering distributed along the PM fiber and by localized reflection from the FLM. When the pump-induced Raman gain becomes higher than the round trip losses in such half-open cavity, the RRFL starts to lase. The output laser power and spectra are measured by a power meter and an optical spectrum analyzer (OSA) Yokogawa AQ6370, respectively. The polarization properties of the generated radiation are investigated with the measurement scheme based on the Glan-Thompson polarizer and polarimeter, see Methods section for more details. As we use linearly polarized pumping whose axis coincides with a chosen (slow) axis of the PM fiber, the Raman gain for another polarization component is strongly discriminated and generation of one (slow) linearly polarized component is expected, similar to conventional Raman fiber lasers with polarized pumping^31^,^32^.

**Figure 2:**
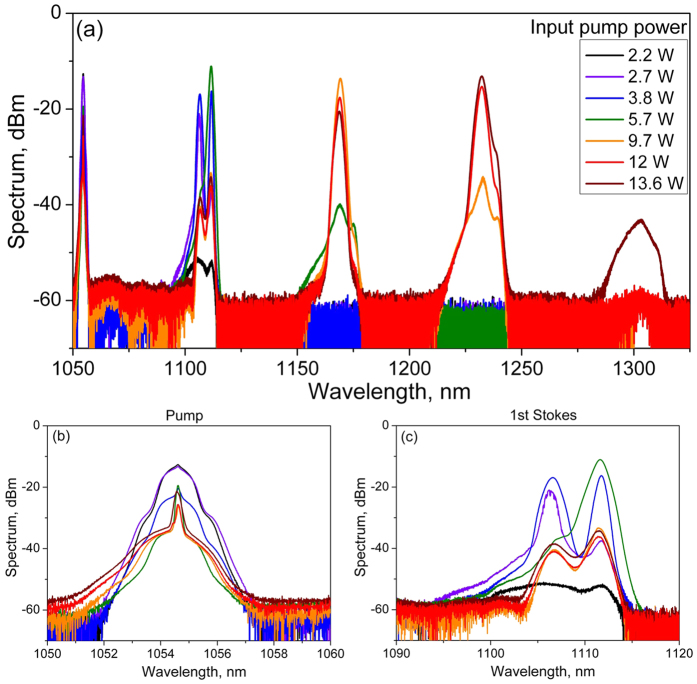
Measured output spectra of the cascaded PM RRFL at different input pump power at 1.05 μm: (**a**) Wide spectral range; (**b**) Transmitted pump; (**c**) 1^st^ Stokes wave.

In the experiment, cascaded random lasing is observed starting from the 1^st^ (1.11 μm) to higher Stokes orders, which appear consecutively in the output spectrum (Fig. 2) with increasing input pump power measured ahead of the 1-km-long PM fiber. The Stokes lines are stable and smooth, in contrast to those in the random fiber laser based on a non-PM fiber with linearly polarized pumping, where random sharp peaks in the output spectra of the 1^st^ Stokes component are observed^28^.

**Figure 3:**
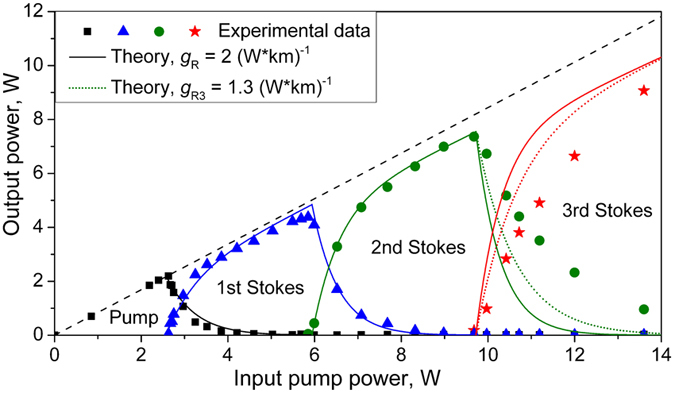
Output power of the cascaded PM RRFL as a function of input pump power. Points correspond to experimental data for transmitted pump (squares) and generated 1^st^ Stokes (triangles), 2^nd^ Stokes (circles) and 3^rd^ Stokes (stars) orders. Solid and dotted lines show the analytical model of power distribution calculated at *g*_R_ = 2 and *g*_R_ = *g*_R3_ = 1.3 (W*km)^−1^, respectively.

Figure 3 shows individual power data for the residual pump (squares), 1^st^ Stokes (triangles), 2^nd^ Stokes (circles) and 3^rd^ Stokes (stars) orders at the output as functions of the input pump power. Only the residual pump wave slightly (by ~15%) attenuated in the 1-km passive fiber is present at the fiber output below the lasing threshold. Its power grows proportionally to the input pump power up to the 1^st^ Stokes threshold (2.6 W). Then the 1^st^ Stokes power starts to grow and the output pump power is depleted as long as almost all the pump power is converted into the Raman lasing. The 1^st^ Stokes power grows up to the 2^nd^ Stokes threshold (5.9 W) and then starts to deplete, and so on for the higher Stokes orders. The maximum input pump power (*P*_*in*_ = 13.6 W) almost corresponds to the 4^th^ Stokes wave threshold revealing a rather low output power (~0.02 W). The absolute optical efficiency of pump-to-Stokes conversion calculated as a ratio of the corresponding *j-*order Stokes power *P*_*Sj*_ (*j* = 1, 2, 3) to the input pump power *P*_*in*_ exceeds 75% for the first and second Stokes waves and approaches 70% for the third Stokes line. Those values are close to the corresponding quantum limits (95, 90 and 86%, respectively) and set record values for the 2^nd^ and the 3^rd^ Raman Stokes waves in random fiber lasers, which are only slightly lower than the maximum efficiency demonstrated for the 1^st^ order (around 88%)^19^,^30^. The generated power is also high amounting to 4.4 W, 7.4 W and 9.1 W for *j* = 1,2,3, respectively. Note that the output power in RRFLs with a half-open cavity is only weakly dependent on the FLM reflectivity^33^, because the Stokes power at the terminating mirror (and its loss accordingly) is lower by several orders of magnitude than the power at the output end^15^,^18^. Therefore, we have high efficiency for all components in spite of reduced reflectivity of the end mirror for higher orders.

**Figure 4:**
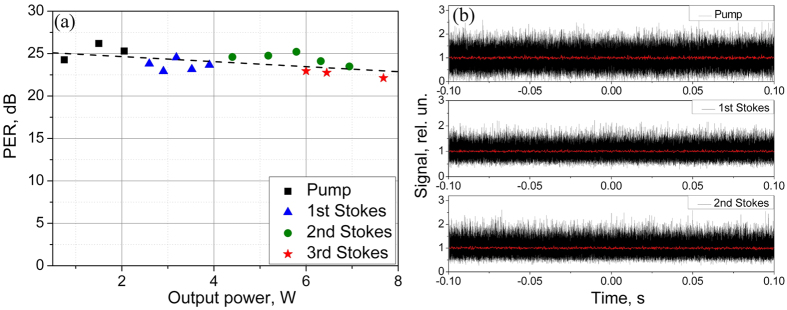
(**a**) Polarization extinction ratio (PER) of the transmitted pump (squares), 1^st^ Stokes (triangles), 2^nd^ Stokes (circles) and 3^rd^ Stokes (stars) orders as a function of their power. Dashed line is RMS fitting. (**b**) Intensity dynamics of the pump, the 1^st^ and the 2^nd^ Stokes waves measured with 400 ps resolution. Red line shows the intensity dynamics averaged over 0.2 ms. It is normalized to unity.

Figure 4a shows the measured polarization extinction ratio of the transmitted pump power and generated Stokes lines. It appears that the PER values of the pump and all Stokes waves are nearly the same, covering 22–26 dB range with a slight decrease in the average PER value with increasing power (see the linear RMS fit shown by the dashed line). Thus, the polarization of the cascaded PM RRFL does not degrade with the generated power and Stokes order. Moreover, the intensity of the output Stokes order is quite stable in the time scale longer than 0.1 ms (see the averaged intensity dynamics in Fig. 4b) being fully stochastic in the scale shorter than 1 ns in accordance with the generated RRFL spectrum consisting of random frequencies with random phases of the Gaussian statistics^20^,^34^.

The linear polarization state of the components principally changes the power evolution of the generated spectra as compared to that for depolarized radiation^20^. As the Raman gain profile in germanosilicate fibers has two nearly equal peaks shifted by ~440 and ~490 cm^−1^ relative to the pump^14^, the 1^st^ Stokes output spectrum consists of two generation lines at 1106 and 1111 nm, accordingly. The distribution between the lines is appreciably changed with an increase in the generated power^14^,^17^,^28^,^33^: at low powers the first line is mainly generated, whereas the second one becomes dominating at high powers (see Fig. 2с). The situation becomes different for the higher Stokes orders, for which the first peak is always dominating. A probable reason is that the Stokes wave playing a role of the pump for the next order has sufficiently larger linewidth as compared with the YDFL pump, which leads to the smoothing of the relatively narrow second peak in the Raman gain spectrum (see the lowest spectrum in Fig. 2c characterizing the amplified spontaneous emission).

**Figure 5:**
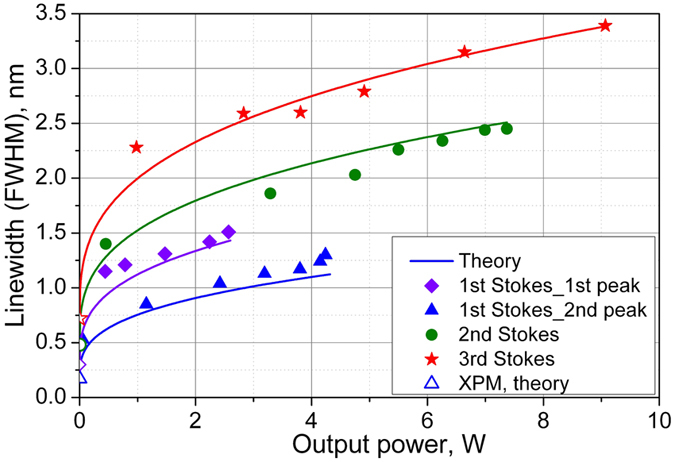
Spectral widths (FWHM) of the individual Stokes line as a function of its power. Points correspond to experimental data for the 1^st^ Stokes wave generated at 440 cm^−1^ (diamonds) and 490 cm^−1^ (triangles) Raman shift, 2^nd^ Stokes (circles) and 3^rd^ Stokes (stars) components. Solid lines show the analytical model of spectral bandwidth.

The linewidth of the first peak is plotted for all Stokes orders as a function of the power in the corresponding line (Fig. 5). In addition, the linewidth of the second peak is shown for the first Stokes wave in its power domain. All spectral lines behave similarly exhibiting the Schawlow-Townes narrowing near the generation threshold and slight broadening with increasing generation power. The generation linewidth for the 440-cm^−1^ Raman peak varies in the range of 1.1–1.5 nm, 1.4–2.5 nm and 2.3–3.4 nm for the 1^st^, 2^nd^ and 3^rd^ Stokes components, correspondingly, whereas the 490-cm^−1^ peak for the 1^st^ Stokes wave is considerably narrower (0.5–1.2 nm). The evolution of the spectral linewidth comprising rather large constant value at the threshold and small power-variable part is principally different from theory^20^. This difference becomes especially evident for the higher Stokes orders.

To explain these rather unique features of the cascaded PM random fiber laser, we developed an analytical theory, which is described in the next section.

### Analytical model

Let us consider the Stimulated Raman Scattering (SRS) process for inelastic scattering of the pump electromagnetic wave with a power *P*_0_ into the *j*-th order Stokes electromagnetic wave at a wavelength *λ*_j_ with a power *P*_j_ within the framework of the balance model described in the Methods section. Under assumptions of equal attenuation for all waves and special relations for their Raman gain coefficients, we succeeded in deriving analytical solutions for their power distributions *P*_j_(*x*). The output power *P*_j_(*L*) of the generated *j*-th order Stokes wave is expressed as


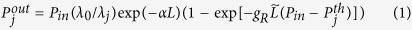


Here *λ*_0_ and *P*_in_ are the wavelength and input power of the pump wave, respectively, *α* is the average attenuation coefficient, *L* and 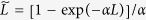 are the total and effective fiber lengths, respectively, *g*_*R*_ is the Raman gain coefficient of the 1st Stokes wave which is set for higher orders too, and 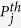 is the power threshold for the *j*-th Stokes wave 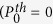 as there is no threshold for the pump wave). The output power of the generated Stokes wave is exponentially approaching the maximum value of the input pump power (if *j* = 1) or of the previous Stokes component playing the role of a pump at *j* > 1. Herewith, the power of the pump wave (or previous Stokes component) starts to decrease exponentially with increasing input pump power above the threshold:


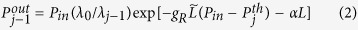


The power curves (solid lines) calculated from Eqs (1, 2) are compared in Fig. 3 with the experimental data for the output pump and Stokes waves of the 1^st^, 2^nd^, and 3^th^ orders. The experimental values for the threshold powers and parameters *L* = 1 km, *α* = 0.15 km^−1^, and *g*_*R*_ = 2 W^−1^*km^−1^ are used in the calculations.

A comparison shows that the derived formulae agree quite well with the experimental data for the first and second orders of the cascaded Raman generation. The difference becomes noticeable for the 3^rd^-order generation whose wavelength is longer by 17% than that for the input pump. The model parameters start deviating noticeably from the experimental ones. If we replace the model gain coefficient *g*_*R*_ in the formula for the 3^rd^-order output power by the real value ( *g*_*R3*_ = 1.3 W^−1^*km^−1^), the agreement between the theory and experiment becomes appreciably better. Moreover, this parameter affects only the intermediate power domain whereas the maximum generated power remains unchanged. It is clear that the power growth above the threshold and the corresponding depletion of previous component are adequately described by the exponential functions predicted by the theory for all orders.

**Figure 6:**
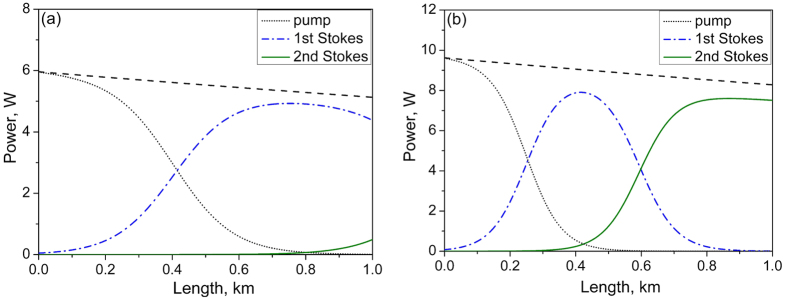
Longitudinal power distribution for different components calculated at the input pump power of 6 W (**a**) and 9.7 W (**b**). Dashed line shows attenuation of the pump power along the fiber.

The longitudinal power distributions calculated from Eqs (10, 11, 12) of Methods section are shown in Fig. 6 for two values of the input pump power *P*_in_ = 6 and 9.7 W, which nearly correspond to the maximum output of the 1^st^ and 2^nd^ Stokes waves. It is seen that the transition regions in the power distribution are described by the steep hyperbolic tangent function predicted by the theory, and different waves are almost fully separated in space. The pump distribution is concentrated near the left end (*x* = 0), whereas the output Stokes order is at the right end (*x* = *L*), and intermediate ones are in between. The output order number is defined only by the available pump power and fiber length.

To obtain the spectral characteristics of the cascaded Raman generation with the Rayleigh feedback, one should treat kinetic equations^20^,^35^ describing the effect of self-phase modulation (SPM) for the generated wave. To obtain the SPM linewidth for the *j*-th Stokes order the kinetic model was modified accordingly taking into account that the experiment is characterized by a relatively large group velocity dispersion as compared with the integral nonlinearity and Raman gain, see Methods section for details. The resulting FWHM linewidth is expressed as a cubic-root function of the output power of the *j*-th Stokes wave *P*_j_^out^:


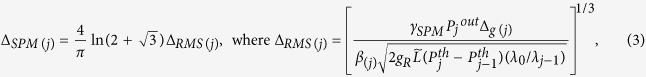


which is supplemented by the pump-induced cross-phase modulation (XPM) effect. The XPM contribution can be estimated at the corresponding threshold, similar to the case of the 1^st^ Stokes wave generation in RFLs^20^,^36^:


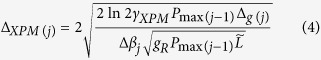


Here *γ*_SPM_ and *γ*_XPM_ are the Kerr nonlinearity coefficients for the SPM and XPM processes, *β*_j_ and Δ*β*_j_ are the second-order dispersion and dispersion walk-off coefficients, respectively, *P*_max(j−1)_ is the maximum power of the (*j* − 1)-th order Stokes wave (or pump wave if *j* = 1) corresponding to the *j*-th Stokes generation threshold, and Δ_g(j)_ is the HWHM width of the Raman gain spectral peak. The solid curves in Fig. 5 show the total linewidth ∆_*FWHM*_ = ∆_*SPM*_ + ∆_*XPM*_ calculated with the nonlinear coefficients *γ*_SPM_ = *γ*_XPM_/2 = 6 W^−1^*km^−1^ and experimental values for the power and Raman widths Δ_g(* j*)_ estimated from the ASE spectrum at low powers (see Fig. 2), which are collected in Table 1 together with the dispersion coefficients for all orders. As a result, we obtain very good agreement of these formulae with the experiment.

## Discussion

The experiment and calculations show that the XPM effect arising from the pump (or previous Stokes order) defines the minimum bandwidth of the generated spectrum amounting to 0.17–0.3, 0.48, 0.72 and 1.03 for the 1st, 2nd, 3rd and 4th Stokes waves, respectively, growing nearly proportionally to the pump power at corresponding threshold. At the same time, the SPM linewidth is a slowly-growing cubic root function of the generated power for all orders. This is quite different from the results obtained for the 1^st^ Stokes generation under conditions of weak dispersion and nonlinearity as compared to the Raman gain, when the linewidth grows almost linearly with generated power^20^. In our case, the linewidth is nearly constant in the broad range of generated powers, but is increasing proportionally to the Stokes order. Another principal difference is introduced by the presence of only one polarization component in contrast to the randomly polarized radiation^20^, for which nonlinear broadening involves additional cross-phase modulation between different polarization components with a twice higher nonlinear coefficient. As a result of elimination of this effect for linear polarization, the absolute linewidth values become sufficiently lower. The spectral properties of the studied RRFL are also different from the case of conventional Raman fiber lasers, where the minimum spectral width is defined by the FBG (or other filtering element) bandwidth^31^,^32^,^36^, whereas its power broadening behaves as a linear or square-root function of the generated power depending on the ratio between nonlinearity and dispersion^36^. Due to the weak broadening for all the orders of cascaded generation in polarized RRFLs, the laser linewidth is narrower than the corresponding Raman gain spectra, because the Schawlow-Townes narrowing is not surpassed by the nonlinear broadening in the entire power range of the corresponding Stokes order. This means that generation of the next-order Stokes wave starts earlier than the linewidth under nonlinear broadening reaches the Raman gain bandwidth, so the developed linearly polarized cascaded RRFL remains to be a laser source distinguishable from an ASE source for all powers and all Stokes orders.

Another interesting spectral effect observed in the studied laser is the reduction of the linewidth for transmitted pump radiation in the regime of its strong depletion by the cascaded Raman generation (see Fig. 2b). Taking that the Raman conversion predominantly occurs for high-intensity peaks existing in the stochastic time-domain trace (see Fig. 4b), which correspond to the broad spectral wings of the integral spectrum, the two-scale pump wave spectrum is differently affected by the Raman process. So, the broadband spectral tails acquired by the pump wave during its propagation in the fiber (via SPM) are efficiently converted into the Stokes waves, whereas the rest narrowband input spectrum (~0.1 nm wide) with the minimum intensity fluctuations survives better and becomes dominating at the output fiber end at strong pump depletion (see Fig. 2b). This is only a qualitative explanation, and this effect, which is not directly related to the studied output characteristics of the cascaded Raman lasing, requires a more detailed study.

The observed power/efficiency behavior of the cascaded random RFL is also quite different from that for conventional RFLs, either linearly polarized^31^,^32^, or non-polarized^36^,^37^, exhibiting a nearly linear power growth with limited efficiency because of high losses for intermediate Stokes components in the cavity. The experiment for the RRFL supported by the balance model shows that the generated power above the threshold exponentially approaches the maximum value being close to the quantum limit, independently of the Stokes order, i.e. almost all input pump photons are converted into the highest-order Stokes photons. This feature of the RRFL is defined by the specific power distributions along the fiber, which are characterized by zero intensity of the intermediate components at the fiber ends and by the maximum power of the highest Stokes order at the output (see Fig. 6). This is quite different from RFLs with conventional cavity, where the intermediate components are reflected from the cavity mirrors at the fiber ends thus experiencing losses^37^. The Raman conversion in the RRFL occurs inside the passive fiber, so only the Rayleigh scattering losses are present here for all waves. They are proportional to the fiber length and nearly equal for the pump and low-order Stokes components. So, the maximum quantum efficiency is limited by the fiber transmission amounting to about 0.85 in the studied scheme with a 1-km PM fiber. As a result, almost all transmitted pump photons are converted into the output Stokes order. The experimental values of the quantum efficiency exceed 79%, 83%, and 77% for the 1^st^, 2^nd^ and 3^rd^ Stokes waves, correspondingly. Moreover, 4^th^ order at 1.3 μm appears at 13.6 W pumping. Therefore, conversion of the 1.055-μm pump wave to the 7^th^ Stokes order beyond 1.55 μm with nearly the same efficiency seems feasible in our scheme at the input power of about 26 W. The pump power level will be reduced proportionally for longer fibers at the expense of decreasing transmission/efficiency, but some efficiency compensation is expected from sufficient reduction of the Rayleigh losses reaching the minimum value of ~0.2 dB/km at 1.55 μm, similar to the effect of increasing number of transmitted photons in non-polarized RRFL with a large Raman shift^18^.

Thus, we demonstrated a linearly-polarized cascaded random Raman lasing in a 1-km-long piece of a PM fiber terminated by a fiber loop mirror at one side. The quantum efficiency of converting the pump radiation (1.05 μm) to the output Stokes wave is around 80%, independent of the Stokes order, which is close to the transmission coefficient of the 1-km fiber used. The output power and efficiency of the Stokes components in the cascaded RRFLs with a half-open cavity is almost independent of the reflectivity of the terminating mirror which is different for different Stokes orders. Herewith, the laser exhibits high power stability, and its PER values exceed 22 dB for all generated orders without any polarization controller.

The proposed approach enables high-efficiency generation of high-quality linearly-polarized laser radiation at almost any wavelength (including the telecom range around 1.55 μm) by using existing high-power pump sources near 1 μm. A complete description of the cascaded Raman lasing in fibers realized here (both in theory and experiment) enables development on this base of high-performance devices, which offer wide opportunities for advanced applications, especially in telecommunications and sensing based on fiber optics links, in which all-fiber PM RRFLs can be easily integrated. Linear polarization and a high power at a relatively narrow bandwidth (which may be further reduced and made tunable by insertion of a spectral filter similar to^21^,^22^,^23^) also offers an efficient frequency doubling that will transfer the generated spectrum in visible range (0.5–0.8 μm is feasible), thus enabling implementation of this source in bio-imaging and display technologies.

## Methods

### Pump source

The linearly polarized MOPA pump source consists of a multimode laser-diode pumped ring cavity Yb-doped fiber laser (YDFL) with depolarized output radiation and two polarization maintaining Yb-doped fiber amplifiers (YDFAs). Fiber polarization beam splitter placed behind the YDFL extracts the linearly polarized component of radiation, which is then launched into the YDFAs. As a result, the pump laser generates linearly polarized radiation at 1054.6 nm with the output power up to 15 W in the fundamental transverse mode.

### Polarization measurements

The polarization properties of the pump and RRFL output radiation were measured by means of a special scheme consisting of a lens, broadband attenuator, Glan-Thompson polarizer and free-space polarimeter with a PAN5710IR2 external sensor head (Thorlabs). The lens and attenuator are used to focus and attenuate the output radiation in front of the polarizer. The polarization extinction ratio is defined as PER = 10log(*P*_max_/*P*_min_), where *P*_min_ and *P*_max_ correspond to the minimum and maximum powers transmitted by the polarizer during the rotation of its optical axis. The transmitted power is measured by the polarimeter which provides a large dynamic range (from −60 to 10 dBm) in 1000–1350 nm spectral regions. We do not use any spectrally selective elements in the measurement setup; therefore, the PER value of an individual spectral component is measured when its output power dominates over the others (see Fig. 3).

### Balance model for the power

In the SRS process, every pump quantum with a frequency *ν*_0_ is absorbed by the medium giving birth to the Stokes wave quantum with a frequency *ν*_1_ and the vibration quantum of the medium with a frequency Δ*ν*_v_ =  *ν*_0_ − *ν*_1_, regardless of the initial frequency *ν*_0_. The same process occurs during the Raman scattering of the Stokes wave into the next-order Stokes wave with a frequency *ν*_2_ = *ν*_1_ − Δ*ν*_v_. The balance equations for the cascaded SRS process converting the power of the pump wave (*P*_0_) into the *j*-th order Stokes waves 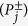, which co(+) and counter(−) propagate with the pump wave along the fiber axis *x,* are written as follows^34^:


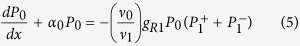



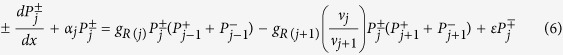


Here *ν*_j_, *α*_j_ and g_R(j)_ are the frequency, attenuation and Raman gain coefficient for *j*-th order Stokes wave, respectively; and *α*_0_ is pump attenuation coefficient. A rather simple analytical solution for the system of differential equations (5, 6) is possible under the following assumption. The pump wave is launched in the fiber at point x = 0, thus *P*_0_(0) = *P*_in_. In addition, the reflecting mirror is also placed at *x* = 0, thus 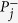
(0) = 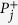
(0). The distributed feedback provided by the Rayleigh backscattering (with coefficient *ε*) of the co-propagating wave (*P*_j_^+^) into the counter-propagating wave (*P*_j_^−^) can be substituted by a local reflector with a reflection coefficient *R*_eff(j)_ ≪ 10^−4^ placed at the point *x* = *L*_RSj_ where *P*_*j*_^+^(*x*) reaches its maximum value:


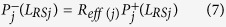


The Rayleigh scattering of the 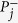 wave into the 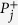 wave can be neglected in comparison with the mirror reflection. In the approximation of point reflectors, 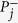 can be expressed via 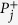 as 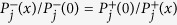. Therefore the counter-propagating wave is much weaker than the co-propagating wave, and the values of *P*_j_^−^ can be neglected in the right side of Eqs (5, 6). In addition, the boundary condition in Eq.(7) is rewritten as 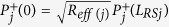. The simplified equations can be integrated under the assumption that the absorption coefficient is independent of the wavelength *α*_0_ = *α*_j_ = *α* and Raman gain coefficient has the model frequency dependence:


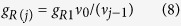


Using this relation, we can express the power threshold for the all Stokes orders obtained from the gain and loss balance in the iteration form with single gain coefficient *g*_*R1*_ = *g*_*R*_:


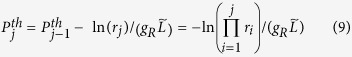


where 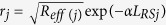.

If the input pump power *P*_in_ exceeds the generation threshold for the *j*-th Stokes wave, the Stokes power distribution along the fiber becomes inhomogeneous:


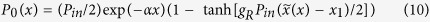



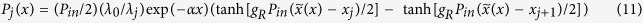



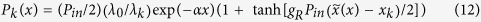


Here *P*_0_(*x*) and *P*_j_(*x*) are the longitudinal distributions involving a specific hyperbolic tangent function for the pump (0) and *j*-th Stokes (*j*) waves at the *k*-th cascade (*j* = 1…*k*); and the coordinate


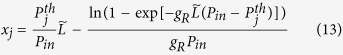


is the point, where 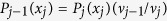. The validity of approximate solutions (10–12) was checked up to the 3rd stage of the cascaded RRFL (see supplementary information), their deviation from exact solutions is negligibly small. Simplified solutions for output power *P*_*j*_^out^ in Eqs (1, 2) of the main text are derived from the solutions in Eqs (10, 11, 12) at *x* = *L* under conditions 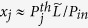 and 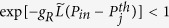.

### Kinetic model for spectra

We assume that the effective dispersion length in the studied RRFL is much larger than the gain length, which allows us to neglect the dispersion effect in the kinetic equations^20^,^35^. Taking the spectral intensity of the Stokes components in the form


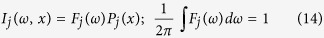


where *ω* is detuning from the line center, and solving equations (A2–A4) from^20^ (see supplementary information), we can write the kinetic equation as


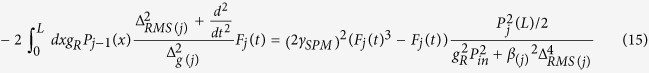


where *γ*_SPM_ is the Kerr nonlinear coefficient for the SPM processes, *β* is the second-order dispersion coefficient, and Δ_RMS_ is the spectral half width. The left side describes spectral filtration over the round trip (by means of the gain spectral function) leading to the Schawlow-Townes spectral narrowing, whereas the right side describes SPM-induced broadening. The solution of this equation is


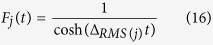


and the corresponding spectral density of the *j*-th Stokes component in the case of *g*_*R*_*P*_in_ ≪ *β*Δ_RMS_^2^ is similar to that for the 1^st^ component under the same condition^35^.


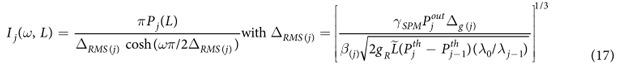


This formula converted to the −3 dB width is given together with the estimated XPM linewidth in Eqs (3 of the main text.

## Additional Information

**How to cite this article**: Babin, S. A. *et al.* High-order random Raman lasing in a PM fiber with ultimate efficiency and narrow bandwidth. *Sci. Rep.* **6**, 22625; doi: 10.1038/srep22625 (2016).

## Supplementary Material

Supplementary Information

## Figures and Tables

**Table 1:** Parameters of the Stokes waves used in the calculations.

J	*λ*, nm	P^th^, W	P_max_, W	β, ps^2^/km	Δβ, ps/m	Δ_g_, *10^12^ rad/s
0	1055	0	2.6			
1 (1^st^ peak)	1106	2.6	2.5	21	1.74	4.8
1 (2^nd^ peak)	1111	2.6	4.4	20.8	1.9	1.7
2	1169	5.9	7.4	17.3	1.43	6.5
3	1232	9.7	9.1	13.5	1.12	7
