# Supplementary material for: High-order random Raman lasing in a PM fiber with ultimate efficiency and narrow bandwidth
Source: Sci Rep. 2016 Mar 4;6:22625. doi: 10.1038/srep22625 (PMC4778055; doi:10.1038/srep22625)
Supplement: Supplementary Information [file srep22625-s1.doc]

**High-order random Raman lasing in a PM fiber with ultimate efficiency and narrow bandwidth: Supplementary Materials**

**Sergey A.Babin, Ekaterina A. Zlobina, Sergey I. Kablukov, and Evgeniy V.Podivilov**

**1. Modified balance equations**

Under the assumptions described in the main text and the following substitutions

$P_{0}(x)=\nu_{0}N_{0}(x)\exp(-\alpha x)$, (S1.1)

$P_{j}^{+}(x)=\nu_{j}N_{j}(x)\exp(-\alpha x)$, (S1.2)

$g_{R(j)}=g/(\nu_{j-1})=g_{R}\nu_{0}/(\nu_{j-1})$ (S1.3)

the balance equations (Eqs. 5 and 6 of the Method Section) may be re-written in the form of integrable system of nonlinear equations describing *j*-th Stokes wave power of the Raman lasing cascade consisting of *k* components (*j* = 1...*k*):

$\frac{dN_{0}}{d\tilde{x}}=-gN_{0}N_{1}$, (S1.4)

$\frac{dN_{j}}{d\tilde{x}}=gN_{j}(N_{j-1}-N_{j+1})$, (S1.5)

$\frac{dN_{k}}{d\tilde{x}}=gN_{k}N_{k-1}$, (S1.6)

where $\tilde{x}(x)=\frac{1-\exp(-\alpha x)}{\alpha}$. Corresponding effective photon numbers *N_j_* satisfy to the following boundary conditions involving effective reflection coefficient *r_j_* for the *j*-th Stokes order:

$N_{0}(0)=P_{in}/\nu_{0}$, (S1.7)

$N_{j}(0)=r_{j}N_{j}(L_{RSj}),\text{ }j=1...k$, (S1.8)

$r_{j}=\sqrt{R_{eff(j)}}\exp(-\alpha L_{RSj})<<1\text{ }$ (S1.9)

**2. Generation of the 1st Stokes wave**

Up to the generation threshold, there exists just the pump wave inducing distributed Raman gain at the 1^st^ Stokes wavelength. The generation threshold for the 1^st^ Stokes wave is defined by the following conditions:

$gN_{0}^{th1}\tilde{L}=-\ln(r_{1})$, (S2.1)

$P_{1}^{th}=-\ln(r_{1})/(g_{R}\tilde{L})$ (S2.2)

Above the threshold,$P_{in}>P_{1}^{th}$, the power distribution along the fiber becomes inhomogeneous for both the waves, and corresponding solution of two-equation system (S.1.4),(S.1.6) at k=1 is:

$N_{0}(x)=\frac{N_{c}}{2}(1-\tanh\left[N_{c}g(\tilde{x}(x)-x_{1})/2\right])$, (S2.3)

$N_{1}(x)=\frac{N_{c}}{2}(1+\tanh\left[N_{c}g(\tilde{x}(x)-x_{1})/2\right])$ , (S2.4)

$N_{0}(0)=N_{c}\frac{1-r_{1}}{1-\exp(-N_{c}g\tilde{L})}$ , (S2.5)

$x_{1}=\frac{1}{N_{c}g}\ln\left(\frac{1-r_{1}}{r_{1}-\exp(-N_{c}g\tilde{L})}\right)$ (S2.6)

Here *N*_c_ = *N*_0_ + *N*_1_ is the total photon number, *x*_1_ is the coordinate at which *N*_0_(*x*_1_) = *N*_1_(*x*_1_). At *r*_1_ << 1, *N*_c_ сan be expressed with a high accuracy via the input pump power:

$N_{c}\approx N_{0}(0)\approx P_{in}/\nu_{0}$, (S2.7)

$x_{1}\approx\frac{P_{1}^{th}}{P_{in}}\tilde{L}-\frac{1}{g_{R}P_{in}}\ln(1-\exp\left[-g_{R}\tilde{L}(P_{in}-P_{1}^{th})\right])$ (S2.8)

Taking into consideration substitution (S1.1)-(S1.3) and condition *N*_1_(0)<<*N*_0_(0), one can obtain a high-accuracy approximation of exact solutions (S2.3)-(S2.6) for longitudinal power distributions in the following form:

$P_{0}(x)=\frac{P_{in}}{2}\exp(-\alpha x)(1-\tanh\left[g_{R}P_{in}(\tilde{x}(x)-x_{1})/2\right])$, (S2.9)

$P_{1}(x)=\frac{P_{in}}{2}(\nu_{1}/\nu_{0})\exp(-\alpha x)(1+\tanh\left[g_{R}P_{in}(\tilde{x}(x)-x_{1})/2\right])$ (S2.10)

The output pump power is exponentially reducing above the threshold:

$\frac{P_{0}^{out}}{P_{in}}=\frac{N_{0}(L)}{N_{0}(0)}\exp(-\alpha L)\approx\exp\left[-g_{R}\tilde{L}(P_{in}-P_{1}^{th})-\alpha L\right]$ (S2.11)

Herewith, the output power for the 1^st^ Stokes wave increases rapidly up to the maximum value being close to the input pump power:

$\frac{P_{1}^{out}}{P_{in}}=\left(\nu_{1}/\nu_{0}\right)\frac{N_{1}(L)}{N_{0}(0)}\exp(-\alpha L)=\left(\nu_{1}/\nu_{0}\right)\exp(-\alpha L)(1-\exp\left[-g_{R}\tilde{L}(P_{in}-P_{1}^{th})\right])$ (S2.12)

The integral of pump power over length becomes “frozen” at the threshold level, whereas the output Stokes wave exhibits linear growth:

$\int_{0}^{L} P_{0}(x)dx=-\ln(r_{1})/g_{R}=P_{1}^{th}\tilde{L}$, (S2.13)

$\int_{0}^{L} P_{1}(x)dx=\left(\nu_{1}/\nu_{0}\right)\ln(N_{0}(0)/N_{0}(L))/g_{R}=\left(\nu_{1}/\nu_{0}\right)(P_{in}-P_{1}^{th})\tilde{L}$ (S2.14)

**3. Generation of the 2nd Stokes wave**

The generation threshold for the 2^nd^ Stokes wave is defined by the condition of its gain to loss balance:

$-\ln(r_{2})=g_{R}\tilde{L}(P_{2}^{th}-P_{1}^{th})$ , (S3.1)

from which the generation threshold is derived:

$P_{2}^{th}=-\ln(r_{1}r_{2})/(g_{R}\tilde{L})$ (S3.2)

Longitudinal power distributions for all the waves can be found in a similar way as for the case of the 1^st^ Stokes wave generation described above. Solving the system of three nonlinear differential equations (S.1.4),(S.1.5),(S.1.6) at *k*=2 with corresponding boundary conditions and $N_{2}\left(0\right)\text{ }=N_{2}(L)r_{2}$, where point *x*_m1_ = (*x*_1_ + *x*_2_)/2 corresponds to the maximum value of *N*_1_(*x*), we can derive cumbersome exact expressions. They can be simplified at *N*_1_(0),*N*_2_(0)<<*N*_0_(0) resulting in approximate solutions for power distributions above the second threshold:

$P_{0}(x)=\frac{P_{in}}{2}\exp(-\alpha x)(1-\tanh\left[g_{R}P_{in}(\tilde{x}(x)-x_{1})/2\right])$, (S3.3)

$P_{1}(x)=\frac{P_{in}}{2}\left(\nu_{1}/\nu_{0}\right)\exp(-\alpha x)(\tanh\left[g_{R}P_{in}(\tilde{x}(x)-x_{1})/2\right]-\tanh\left[g_{R}P_{in}(\tilde{x}(x)-x_{2})/2\right])$, (S3.4)

$P_{2}(x)=\frac{P_{in}}{2}\left(\nu_{2}/\nu_{0}\right)\exp(-\alpha x)(1+\tanh\left[g_{R}P_{in}(\tilde{x}(x)-x_{2})/2\right])$ (S3.5)

Here

$x_{1}\approx\frac{P_{1}^{th}}{P_{in}}\tilde{L}$ , $x_{2}\approx\frac{P_{2}^{th}}{P_{in}}\tilde{L}-\frac{\ln(1-\exp\left[-g_{R}\tilde{L}(P_{in}-P_{2}^{th})\right])}{g_{R}P_{in}}$ (S3.6)

At *x*<*x*_1_, the pump power is the highest, whereas the 1^st^ and 2^nd^ Stokes powers dominate at *x*_1_ <*x* <*x*_2_, and *x* > *x*_2_, respectively.

Above the second threshold, the output pump power starts growing at rather low level:

$\frac{P_{0}^{out}}{P_{in}}=\frac{N_{0}(L)}{N_{0}(0)}\exp(-\alpha L)=r_{2}\exp(-\alpha L)$ (S3.7)

Herewith, the 1^st^ Stokes output power is exponentially reducing:

$\frac{P_{1}^{out}}{P_{in}}\approx\left(\nu_{1}/\nu_{0}\right)\exp\left[-g_{R}\tilde{L}(P_{in}-P_{2}^{th})-\alpha L\right]$ (S3.8)

The output power for the 2^nd^ Stokes wave increases rapidly up to the maximum value being close to the input pump power:

$\frac{P_{2}^{out}}{P_{in}}=\left(\nu_{2}/\nu_{0}\right)\frac{N_{2}(L)}{N_{0}(0)}\exp(-\alpha L)\approx\left(\nu_{2}/\nu_{0}\right)\exp(-\alpha L)\left(1-\exp\left[-g_{R}\tilde{L}(P_{in}-P_{2}^{th})\right]\right)$ (S3.9)

The integral of pump power (and 1^st^ Stokes wave) over length are “frozen” at the threshold level:

$\int_{0}^{L} P_{0}(x)dx=P_{1}^{th}\tilde{L}=-\ln(r_{1})/g_{R}$, (S3.10)

$\int_{0}^{L} P_{1}(x)dx\approx\left(\nu_{1}/\nu_{0}\right)(P_{2}^{th}-P_{1}^{th})\tilde{L}=-\left(\nu_{1}/\nu_{0}\right)\ln(r_{2})/g_{R}$, (S3.11)

whereas the 2^nd^ Stokes wave exhibits linear growth:

$\int_{0}^{L} P_{2}(x)dx\approx\left(\nu_{2}/\nu_{0}\right)(P_{in}-P_{2}^{th})\tilde{L}$ (S3.12)

**4. Generation of the 3rd Stokes wave**

The generation threshold for the 3^rd^ Stokes wave is defined by the condition of its gain to loss balance:

$P_{3}^{th}=P_{2}^{th}-\ln(r_{3})/(g_{R}\tilde{L})=-\ln(r_{1}r_{2}r_{3})/(g_{R}\tilde{L})$ (S4.1)

To find longitudinal power distributions for all the waves, one should solve the system of four nonlinear differential equations (S.1.4)-(S.1.6) at *k*=3

with the corresponding boundary conditions $\frac{N_{1}(0)}{N_{1}(x_{m1})}=r_{1},\text{ }$ $\frac{N_{2}(0)}{N_{2}(x_{m2})}=r_{2},\text{ }$ $\frac{N_{3}(0)}{N_{3}(L)}=r_{3},\text{ }$ where *x*_m1_, *x*_m2_ are the maximum points of waves *N*_1,2_(*x*).

In this case, cumbersome exact solutions can be also simplified at conditions *N*_1_(0), *N*_2_(0), *N*_3_(0) << *N*_0_(0) resulting in approximate equations for the longitudinal power distributions above the third threshold in the following form:

$P_{0}(x)=\frac{P_{in}}{2}\exp(-\alpha x)(1-\tanh\left[g_{R}P_{in}(\tilde{x}(x)-x_{1})/2\right])$, (S4.2)

$P_{1}(x)=\frac{P_{in}}{2}\left(\nu_{1}/\nu_{0}\right)\exp(-\alpha x)(\tanh\left[g_{R}P_{in}(\tilde{x}(x)-x_{1})/2\right]-\tanh\left[g_{R}P_{in}(\tilde{x}(x)-x_{2})/2\right])$, (S4.3)

$P_{2}(x)=\frac{P_{in}}{2}\left(\nu_{2}/\nu_{0}\right)\exp(-\alpha x)(\tanh\left[g_{R}P_{in}(\tilde{x}(x)-x_{2})/2\right]-\tanh\left[g_{R}P_{in}(\tilde{x}(x)-x_{3})/2\right])$, (S4.4)

$P_{3}(x)=\frac{P_{in}}{2}\left(\nu_{3}/\nu_{0}\right)\exp(-\alpha x)(1+\tanh\left[g_{R}P_{in}(\tilde{x}(x)-x_{3})/2\right])$ (S4.5)

Here

$x_{1}\approx\frac{P_{1}^{th}}{P_{in}}\tilde{L}$, $x_{2}\approx\frac{P_{2}^{th}}{P_{in}}\tilde{L}$, $x_{3}\approx\frac{P_{3}^{th}}{P_{in}}\tilde{L}-\frac{\ln(1-\exp\left[-g_{R}\tilde{L}(P_{in}-P_{3}^{th})\right])}{g_{R}P_{in}}$ (S4.6)

The output powers for the pump and 1^st^ Stokes waves, *P*_0_^out^ and *P*_1_^out^, are very low. The output power for the 2^nd^ Stokes wave *P*_2_^out^ decreases exponentially, whereas 3^rd^ Stokes wave power *P*_3_^out^ increases rapidly:

$P_{2}^{out}\approx P_{in}\left(\nu_{2}/\nu_{0}\right)\exp\left[-g_{R}\tilde{L}(P_{in}-P_{3}^{th})-\alpha L\right]$, (S4.7)

$P_{3}^{out}\approx P_{in}\left(\nu_{3}/\nu_{0}\right)\exp(-\alpha L)\left(1-\exp\left[-g_{R}\tilde{L}(P_{in}-P_{3}^{th})\right]\right)$ (S4.8)

The integral of 2^nd^ Stokes wave over length are “frozen” at the threshold level, whereas the 3^rd^ Stokes power exhibits linear growth at this stage:

$\int_{0}^{L} P_{2}(x)dx\approx\left(\nu_{2}/\nu_{0}\right)(P_{3}^{th}-P_{2}^{th})\tilde{L}$, (S4.9)

$\int_{0}^{L} P_{3}(x)dx\approx\left(\nu_{3}/\nu_{0}\right)(P_{in}-P_{3}^{th})\tilde{L}$ (S4.10)

**5. Simplified kinetic equation**

In this part a derivation of the simplified kinetic equation is present. It is derived from the generalized kinetic equation (see [20, 34]) taking into account that in the studied RRFL the effective dispersion length is much larger than the gain length. Therefore we can neglect by the dispersion effect in the kinetic equations. Taking the spectral intensity of Stokes components in the form

$I_{j}(\omega,x)=F_{j}(\omega)P_{j}(x)$, $\frac{1}{2\pi}\int F_{j}(\omega)d\omega=1$ (S5.1)

the generalized kinetic equation can be rewritten as

(S5.2)

Since *P_j_*(*x*) *∝* exp(*g*_R_*P*_in_*x*) grows as exponent of *g_R_P_in_* reaching maximum value at the output boundary (see previous section), the RHS integral can be evaluated as

$\mathrm{Re}\int^{x} P_{j}(x')\exp(i\beta\omega_{1}\omega_{2}x')dx'\approx\frac{g_{R}P_{in}}{g_{R}^{2}P_{in}^{2}+\beta^{2}\omega_{1}^{2}\omega_{2}^{2}}P_{j}(x)$ (S5.3)

Replace frequency detunings in the denominator by their characteristics values which are equal to a half width, *ω*_1,2_ → Δ_RMS_, and obtain simplified kinetic equation in the form:

(S5.4)

After the round trip integration, we obtain the simplified kinetic equation for correlation function$F_{j}(t)=\frac{1}{2\pi}\int F_{j}(\omega)\exp(i\omega t)d\omega$:

(S5.5)

The solution to this equation is analyzed in Methods section.
